# Hypothalamic Sirt1 protects terminal Schwann cells and neuromuscular junctions from age‐related morphological changes

**DOI:** 10.1111/acel.12776

**Published:** 2018-05-30

**Authors:** Alison K. Snyder‐Warwick, Akiko Satoh, Katherine B. Santosa, Shin‐ichiro Imai, Albina Jablonka‐Shariff

**Affiliations:** ^1^ Division of Plastic Surgery Department of Surgery Washington University School of Medicine St. Louis MO USA; ^2^ Department of Developmental Biology Washington University School of Medicine St. Louis MO USA; ^3^ Sleep and Aging Regulation Research Project Team National Center for Geriatrics and Gerontology Obu Aichi Japan; ^4^ Project for Elucidating and Controlling Mechanisms of Aging and Longevity Japan Agency for Medical Research and Development Tokyo Japan

**Keywords:** aging, neuromuscular junction, NMJ, Sirt1, sirtuin, terminal Schwann cell

## Abstract

Neuromuscular decline occurs with aging. The neuromuscular junction (NMJ), the interface between motor nerve and muscle, also undergoes age‐related changes. Aging effects on the NMJ components—motor nerve terminal, acetylcholine receptors (AChRs), and nonmyelinating terminal Schwann cells (tSCs)—have not been comprehensively evaluated. Sirtuins delay mammalian aging and increase longevity. Increased hypothalamic Sirt1 expression results in more youthful physiology, but the relationship between NMJ morphology and hypothalamic Sirt1 was previously unknown. In wild‐type mice, all NMJ components showed age‐associated morphological changes with ~80% of NMJs displaying abnormalities by 17 months of age. Aged mice with brain‐specific *Sirt1* overexpression (BRASTO) had more youthful NMJ morphologic features compared to controls with increased tSC numbers, increased NMJ innervation, and increased numbers of normal AChRs. Sympathetic NMJ innervation was increased in BRASTO mice. In contrast, hypothalamic‐specific *Sirt1* knockdown led to tSC abnormalities, decreased tSC numbers, and more denervated endplates compared to controls. Our data suggest that hypothalamic Sirt1 functions to protect NMJs in skeletal muscle from age‐related changes via sympathetic innervation.

## 1 INTRODUCTION

Muscle function is essential to overall health, stability, and safe mobility. Aging is associated with progressive skeletal muscle mass decline, muscle fiber denervation, and motor unit loss and remodeling (Santulli & Iaccarino, 2013). With age, degenerative changes may affect not only muscle and nerve, but also the interface between the two, the neuromuscular junction (NMJ). The NMJ is integral to motor function; it is a cholinergic synapse that controls muscle contraction. The NMJ does not have a fixed, permanent structure, but instead shows plasticity in response to muscle injury, disease, and aging. It is composed of three main elements: the motor nerve terminal, acetylcholine receptors (AChRs), and specialized glial cells termed perisynaptic Schwann cells or terminal Schwann cells (tSCs). Unlike the myelinating Schwann cells that surround motor nerve axons, tSCs are nonmyelinating glial cells that surround nerve terminals at the NMJ. tSCs regulate morphological stability and function of the NMJ and play a critical role in regulating the nerve–muscle interface with important roles in synaptic regeneration in homeostasis (Reddy, Koirala, Sugiura, Herrera & Ko, 2003), after injury (Kang, Tian & Thompson, 2003), and in aging (Griffin & Thompson, 2008).

Age‐associated NMJ degeneration is well characterized in animals and humans (Balice‐Gordon, 1997; Boaro, Soares & Konig, 1998; Deschenes, Roby, Eason & Harris, 2010; Jang & Van Remmen, 2010; Kawabuchi et al., 2001; Luff, 1998; McMullen & Andrade, 2009; Smith & Chapman, 1987; Steinbach, 1981; Wernig & Herrera, 1986). Aging results in progressive NMJ degradation causing a steady decline in muscle mass and strength, termed senile muscle atrophy, or sarcopenia. Interactions among tSCs, presynaptic nerve terminals, and postsynaptic endplates and muscle play critical roles in synaptic growth, maintenance, and survival. The exchange of trophic factors has been implicated in pre‐ and postsynaptic development as well as preserving neuronal and synaptic plasticity at the NMJ. In addition, factors such as mitochondrial dysfunction, oxidative stress, inflammation, changes in muscle fiber innervation, and motor unit mechanical properties probably contribute to NMJ degeneration. Interventions such as caloric restriction and exercise may positively affect the NMJ and attenuate the age‐related progressive impairment in motor function (Valdez et al., 2010).

A mammalian sirtuin, Sirt1, controls several age‐associated pathophysiological processes in energy homeostasis, cognitive function, emotion, and neurogenesis (Satoh, Imai & Guarente, 2017). Recent studies have demonstrated that hypothalamic Sirt1 regulates mammalian aging and longevity in mice. Stereotactic injection of *Sirt1*‐expressing lentiviruses into the dorsomedial hypothalamus (DMH) of aged adult wild‐type (WT) mice ameliorates age‐associated declines in physical activity and body temperature, supporting the notion that the hypothalamus, specifically the DMH, is one of the control centers for mammalian aging and longevity (Satoh et al., 2010, 2013). Mice with brain‐specific overexpression of *Sirt1* (BRASTO) show a significant delay in aging with extended lifespans in both males and females (Satoh et al., 2013). Specifically, skeletal muscle in BRASTO mice maintains youthful morphology and mitochondrial function during the aging process due to enhanced sympathetic nervous tone during the dark period. Although the mechanism by which the signal from the hypothalamus is specifically directed to skeletal muscle remains unknown, increased sympathetic tone may contribute to skeletal muscle and NMJ changes and increased longevity to delay the effects of aging.

Sympathetic innervation controls muscle metabolism, maintenance, and function of nerve–muscle contact (Roatta & Farina, 2009). There are a few studies reporting on direct innervation of skeletal muscle fibers by nonmyelinated, noradrenergic fibers (Barker & Saito, 1981; Lynch & Ryall, 2008), suggesting that sympathetic actions on skeletal muscle are at least partially mediated by neural mechanisms. It has been reported that sympathetic neurons coinnervate several targets in muscle, including blood vessels, motor neurons, muscle fibers, and NMJs (Khan et al., 2016; Rudolf et al., 2013). Moreover, cAMP/PKA‐dependent signaling at the NMJ is important for synapse stabilization and metabolic control of AChR function (Li, Yi & Thompson, 2011).

This study examines the effects of aging on all components of the NMJ, including tSCs. In addition, we investigate the impact of differing levels of brain or hypothalamic *Sirt1* expression on NMJ architecture in skeletal muscle and suggest a role for sympathetic innervation in mediating the systemic effects of central *Sirt1* changes at the level of the NMJ.

## 2 RESULTS

### 2.1 Body mass and sternomastoid (SM) mass decrease with age in WT mice

Peak average body weight occurred at 14 months of age (41.34 ± 4.9 g) and declined by 26% by 33 months of age (30.67 ± 3.0 g) in WT mice. Wet weight of the SM muscle in isolation paralleled total body mass with a 33% decline in mass between 14 and 33 months of age (0.021 ± 0.003 g and 0.014 ± 0.001 g, respectively; Table 1).

**Table 1:**
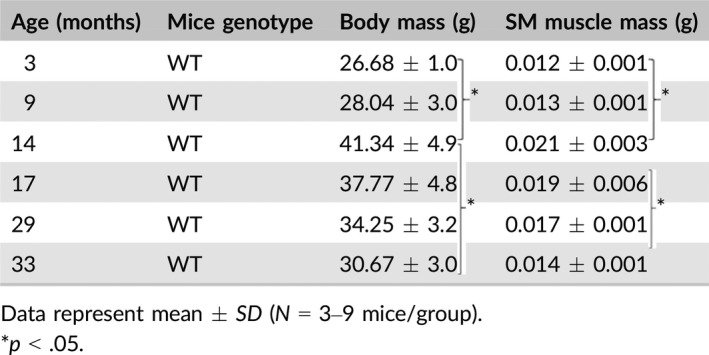
Body weight and SM muscle mass

### 2.2 NMJ morphologic abnormalities increase with age in WT mice

**Figure 1:**
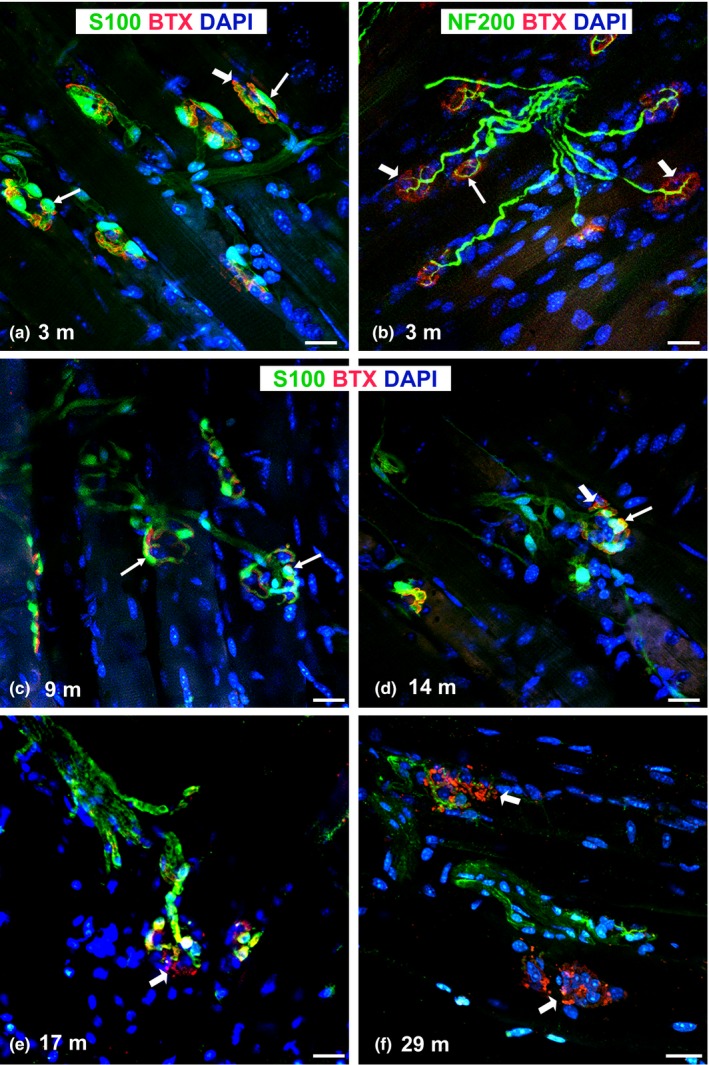
Representative confocal images of NMJ morphology in the sternomastoid muscle of adult WT mice with age. (a–c) Characteristic NMJ morphology is seen in 3‐ and 9‐month‐old mice with clusters of tSCs (*arrows*, a, c) near the nerve terminal (*arrows*, b) and AChR staining (*arrowheads,* b). (d–f) Representative images of NMJs demonstrate less colocalization of tSCs (*arrows*) and AChRs (*arrowheads*) with advancing age. AChRs acquire a fragmented and granular appearance with age (*arrowheads* in e, f). S100 Ab (for SCs); NF200 Ab = antineurofilament antibody; BTX = α‐bungarotoxin (for AChRs); DAPI = nuclear staining; m = months; Scale bar = 20 μm

**Figure 2:**
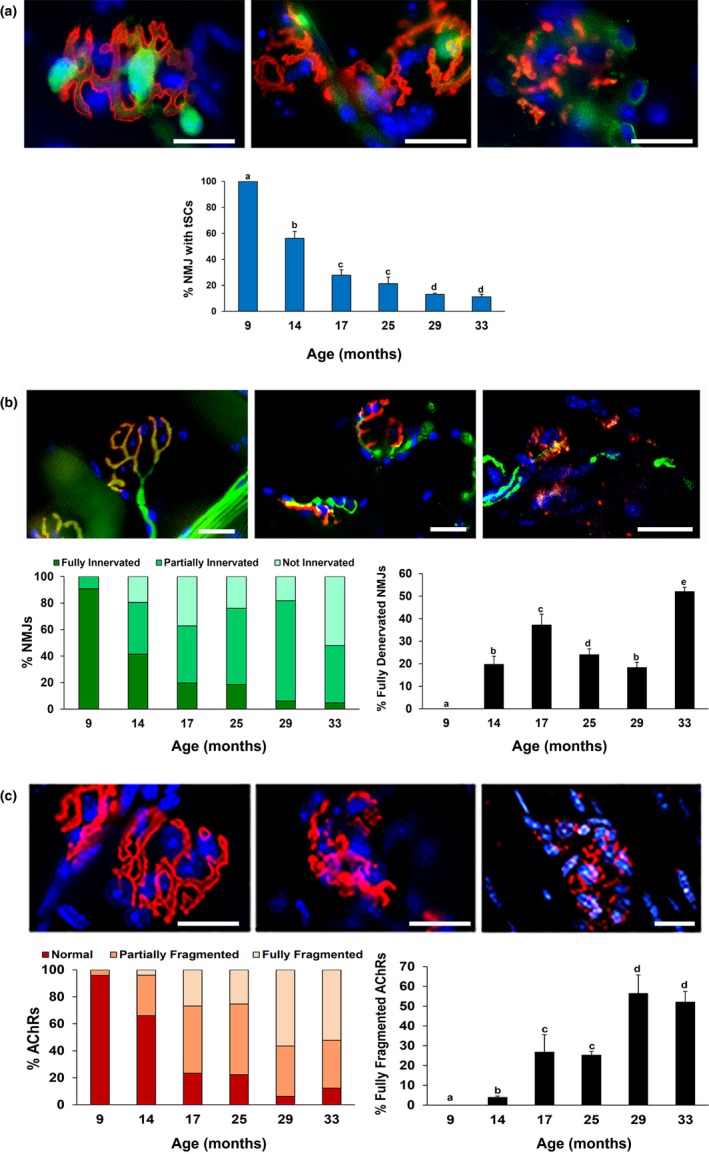
Advancing age results in more frequent NMJ morphological alterations in sternomastoid muscle of WT mice. (a) Top images show decrease in tSC (green) number and staining intensity with age. Graph represents the percentage of NMJs with tSCs. (b) Top images (from left to right side) represent examples of fully, partially, and not innervated (green) NMJs, respectively. Graphs show the proportions of NMJs with the three degrees of innervation (left graph) and the percentage of fully denervated NMJs (right graph). (c) Top images (from left to right side) represent examples of normal, partially, and fully fragmented AChRs (red), respectively. Graphs show the proportion of NMJs with the three classifications of endplate fragmentation (left graph) and the percentage of fully fragmented AChRs (right graph). S100 Ab (for tSCs, a); NF200 Ab (for neurofilaments, b); BTX = α‐bungarotoxin (for AChRs, a–c); DAPI = nuclear staining (blue, a–c); Scale bar = 20 μm. Data ± *SD*; different letter indicates significant difference; *p *<* *.05

Confocal and fluorescent images of tSCs (labeled with S100 Ab), presynaptic nerve terminals (labeled with NF200 and SV2 Abs), and postsynaptic endplates (stained with α‐BTX, which is specific for AChRs) enabled visualization and analyses of NMJs in SM muscles of 3‐, 9‐, 14‐, 17‐, 25‐, 29‐, and 33‐month‐old WT mice. NMJs from mice between 3 and 9 months of age showed normal morphological characteristics including 2–5 tSCs, a single nerve terminal, and branched, pretzel‐like AChR staining at each NMJ (Figure 1a–c). In contrast, NMJ organization was altered in all groups of aged mice from 14 through 33 months of age, with >90% of NMJs demonstrating some type of morphologic abnormality by age 29 months. Aged mice showed coincidental abnormalities of the tSCs, nerve terminals, and endplates (Figures 1d–f and 2), which are described in detail below.

#### 2.2.1 Terminal Schwann cells

The Schwann cell marker S100 was present in tSCs at the NMJ, and these cells had ovoid‐shaped nuclei and cytoplasmic processes that colocalized with AChRs. tSCs in young adult mice were present in almost 100% of NMJs and stayed in contact with the entire endplate (Figures 1 and 2a). In contrast, tSC numbers decreased with age (Figure 1f). The percentage of NMJs with tSCs present decreased significantly (*p *<* *.05) from 100% at 9 months of age to 11.2 ± 1.9% at 33 months of age (Figure 2a). In addition, tSC bodies exhibited irregular contour and variability in staining intensity with S100 Ab (Figure 2a). tSC processes were thinner with advancing age, but overall were less affected by mouse age than tSC bodies (data not shown).

#### 2.2.2 Presynaptic nerve terminal

NMJ innervation was evaluated with immunostaining with NF200 Ab and α‐BTX to identify the presence of nerve terminals. For quantification, NMJs were divided into three groups: (i) fully innervated, (ii) partially innervated (less branching or fragmented), and (iii) not innervated (denervated) (Figure 2b). Nerve terminals from young adults were distinct, branching, and colocalized with endplates (α‐BTX staining) (Figure 1b). NMJ innervation significantly (*p *<* *.05) decreased with age beginning at 14 months of age with denervation of nearly 20% of NMJs (Figure 2b). The percentage of fully denervated NMJs increased to over 35% at 17 months of age, but then decreased again until advanced age (33 months) (Figure 2b). Aged mice demonstrated almost no nerve branching and frequently had no nerve terminal associated with the NMJ. Some nerve sprouting was observed only in 14‐month‐old mice, but by 33 months of age, nearly half of the assessed NMJs were denervated (Figure 2b). Variability in nerve morphology was also seen, including enlarged and spherical nerve ends as well as thin axon terminals (Figure 2b).

#### 2.2.3 Motor endplates (AChRs)

Age‐associated changes on postsynaptic AChRs were characterized after immunostaining with α‐BTX, and the number of AChR fragments per NMJ was evaluated (Figures 1 and 2c). Data are presented as percentage of NMJs that contain normal, partially fragmented, or fully fragmented AChRs. Most endplates from young mice (Figures 1 and 2c) formed normal, pretzel‐shaped, synaptic gutters consisting of a continuous structure with less than five fragments. Endplates from aged mice formed a single cluster (often diffusive) or were more fragmented or granular (Figures 1 and 2c). At 14 months of age, ~34% of NMJs showed partial or full fragmentation which increased drastically between 17 and 33 months of age (*p *<* *.05). By 33 months of age, only ~12% of AChRs showed normal morphology.

### 2.3 Aged BRASTO mice have a more youthful NMJ morphology than WT mice

**Figure 3:**
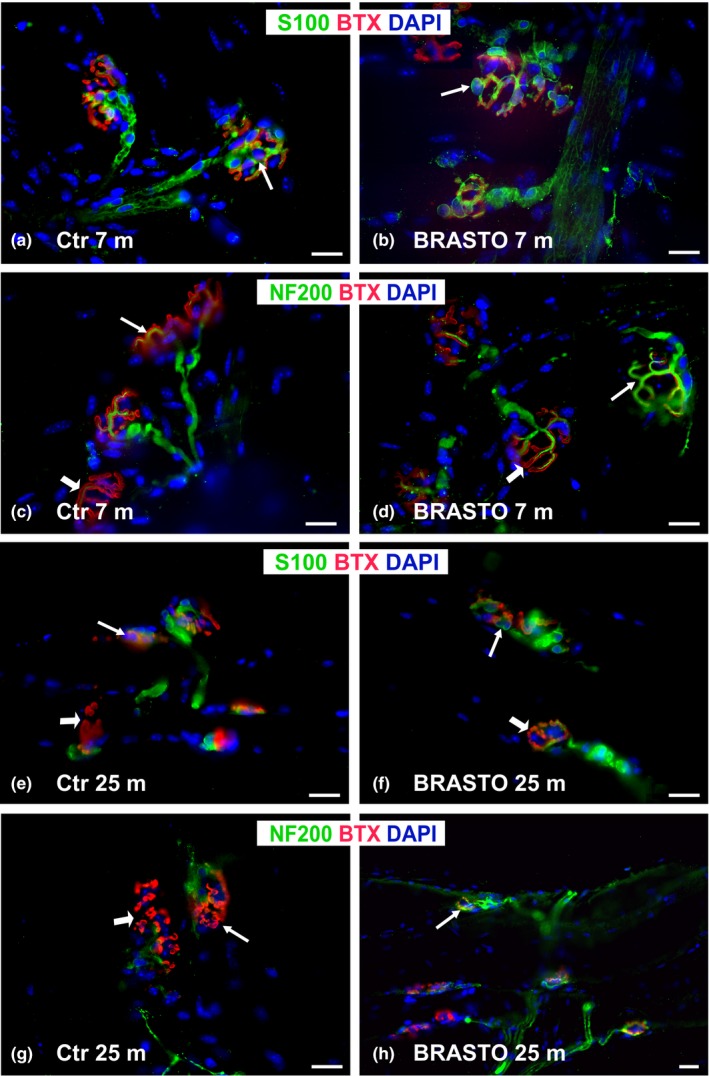
NMJ morphology in BRASTO mice and age‐matched controls. NMJ morphology in young (7‐month‐old) BRASTO mice (b, d) did not differ from characteristic, healthy NMJ morphology seen in age‐matched controls (a, c). Aged (25‐month‐old) BRASTO (f, h) mice have a more youthful NMJ morphology than age‐matched control (Ctr) mice (e, g). NMJs in BRASTO mice have distinct tSCs, clear innervation (*arrow*), and pretzel‐shaped motor endplates (*arrowhead* in f, h) in contrast to the fewer tSCs, incomplete or absent innervation, and fragmented AChRs (*arrowheads* in e, g) of NMJs in Ctr mice. Representative images of NMJs from sternomastoid muscle stained with S100 Ab (for tSCs, green), NF200 Ab (for neurofilaments, green), BTX (for AChRs, red), and DAPI (nuclear staining, blue). Scale bar = 20 μm

**Figure 4:**
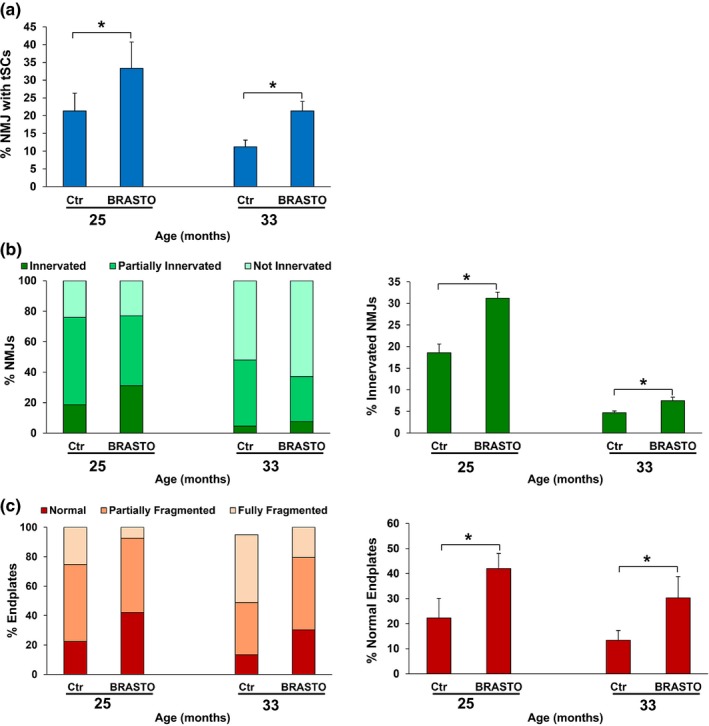
Quantification analyses of NMJs from aged (25‐ and 33‐month‐old) BRASTO mice demonstrate healthier NMJ morphologies compared to age‐matched control (Ctr) mice. (a) BRASTO mice display more NMJs with tSC bodies compared to Ctr at both ages. (b) NMJs from BRASTO mice display less partially innervated with a similar level of denervation (not innervated, left graph) and have greater percentages of full innervation (right graph) than Ctr. (c) Less AChR fragmentation (left graph) and more normal motor endplates, defined as 5 or fewer fragments, are seen in BRASTO mice compared to age‐matched Ctr. Data ± *SD*; *p <* *.05

Each of the three main NMJ components (tSCs, nerve terminal, and AChRs) was compared in young (7‐month‐old) and aged (25‐ and 33‐month‐old) BRASTO mice and age‐matched WT controls qualitatively (Figure 3) and quantitatively (Figure 4). NMJ morphology in young (7‐month‐old) BRASTO mice did not differ from characteristic, healthy NMJ morphology seen in age‐matched controls (Figure 3a–d). When mice became old, more tSC processes and tSC bodies were observed at the NMJ in SM muscles of BRASTO mice compared to controls (Figures 3 and 4a). The percentage of NMJs with tSC bodies present significantly (*p *<* *.05) increased and was nearly double that of controls at 33 months of age (Figure 4a). Similarly, the percentage of fully innervated NMJs was significantly higher in the aged BRASTO SM muscles compared to controls at both ages (*p *<* *.05, Figure 4b). With respect to motor endplates, full fragmentation of the AChRs was significantly lower in BRASTO mice compared to their age‐matched controls (*p* < .05, Figure 4c). Similarly, the percentage of normal motor endplates in BRASTO mice was approximately double that of their age‐matched controls at both 25 and 33 months of age (*p *<* *.05, Figure 4c). In summary, the aged BRASTO mice showed a more youthful morphology of each of the three main NMJ components compared to WT controls.

### 2.4 DMH‐specific Sirt1 knockdown results in a more aged NMJ morphology

**Figure 5:**
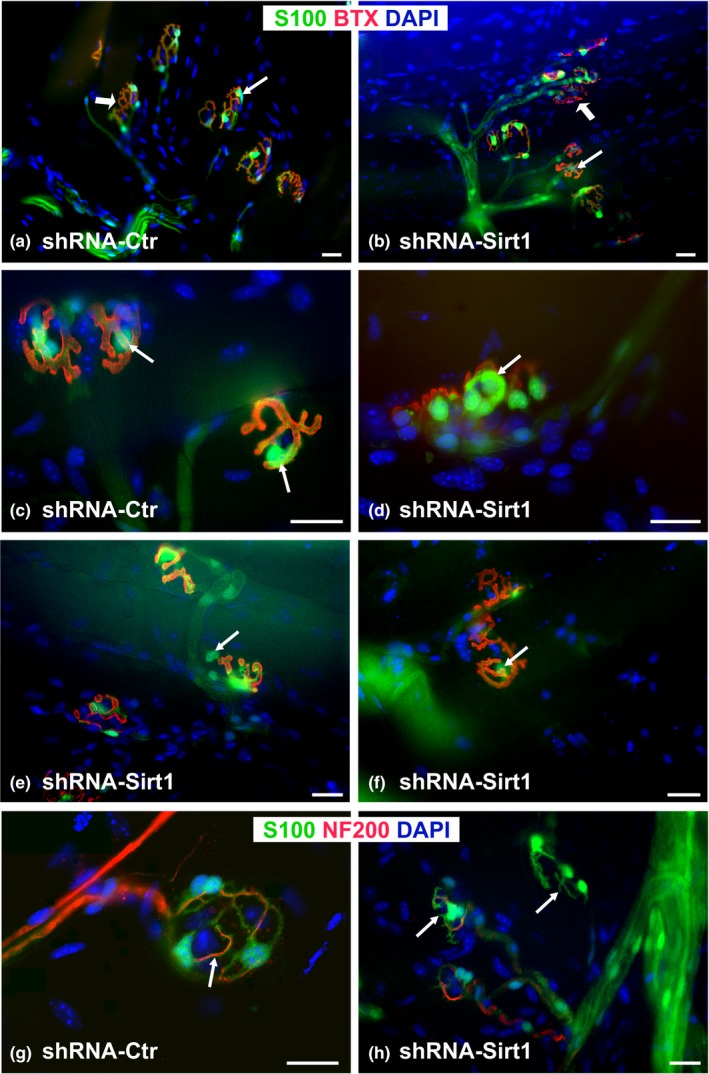
DMH‐specific knockdown of *Sirt1* (shRNA‐*Sirt1*, b, d–f, h) in 3‐month‐old WT mice results in a more aged NMJ morphology compared to age‐matched, control‐injected mice (shRNA‐Ctr, a, c, g). Representative low magnification images of whole mount sternomastoid muscle show tSC abnormalities (less staining intensity and fewer numbers) in shRNA‐*Sirt1* mice (b, e, f). Note also tSC anomalies (*arrows*): large and intensely stained tSC bodies (d), tSCs migrated outside of NMJ area (e), or lightly stained with few processes colocalized with AChRs (f) in shRNA‐*Sirt1* mice. Less severe changes are observed for nerve terminals (h, *arrows*), and almost no changes are seen in motor endplates (b, d–f). S100 Ab (for tSCs, green), NF200 Ab (for neurofilaments, green), BTX (for AChRs, red), and DAPI (nuclear staining, blue). Scale bar = 20 μm

**Figure 6:**
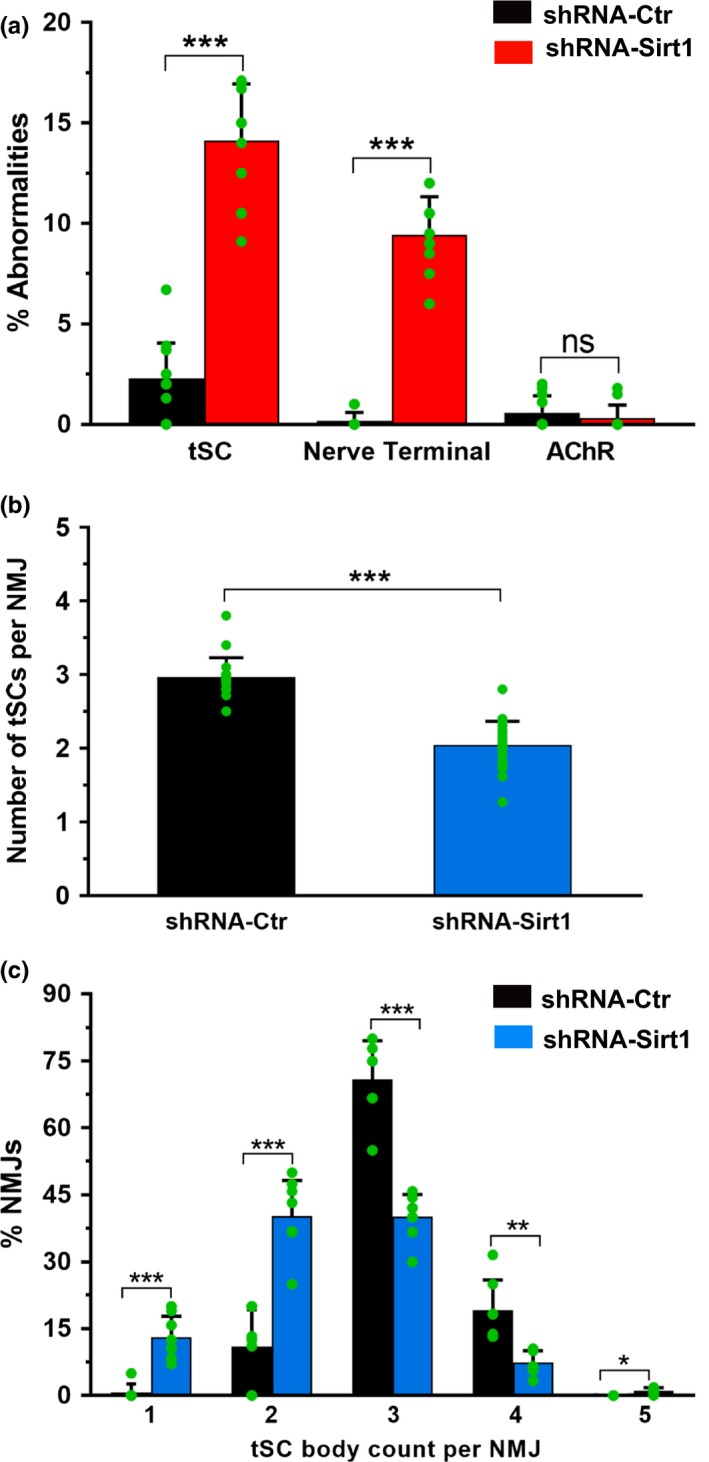
DMH‐specific knockdown of *Sirt1* (shRNA‐*Sirt1*) results in more aged NMJ phenotypes compared to control (shRNA‐Ctr) mice. (a) shRNA‐*Sirt1* mice show significantly greater proportions of abnormalities (one or more) in tSCs and nerve terminals, but not AChRs, compared to controls. (b) shRNA *Sirt1* mice have fewer tSC bodies per NMJ compared to control mice. (c) Similarly, the distribution of tSC numbers per NMJ is decreased compared to controls. Data ± *SD*; each green dot represents average value calculated per muscle; ***p < .001, **p <* *.01, *p < .05, ns=not significant

DMH and lateral hypothalamus (LH) neurons are responsible for phenotypes observed in aged BRASTO mice (Satoh et al., 2013). In particular, Sirt1 in the DMH plays a critical role in maintaining physical activity. NMJ analyses were thus performed 3 weeks after stereotactic injections of shRNA‐*Sirt1* or shRNA‐Ctr into the DMH of 3‐month‐old WT mice. The efficiency of the lentiviral knockdown was noted to be a 65% reduction in mRNA (data not shown) and similar to previous reports (Satoh et al., 2013). DMH‐specific *Sirt1* knockdown accelerated age‐associated NMJ morphologic changes compared to age‐matched controls (Figures 5 and 6). A variety of tSC morphological abnormalities (Figure 5) not observed in normal, healthy NMJs in control mice (13.6 ± 0.74%, *p* < .05; Figure 6a) was also seen in the NMJs from DMH‐specific *Sirt1* knockdown mice. tSCs demonstrated atypically large and intensively stained cell bodies (Figure 5d), often observed outside the NMJ area (Figure 5e), and many were also lightly stained with S100 Ab with some processes (Figure 5f). In addition, the number of tSCs associated with each endplate was less in shRNA‐*Sirt1* mice (Figure 6). On average, NMJs had 2.98 ± 0.11 associated tSCs in shRNA‐Ctr mice, and the majority of NMJs had three or four tSCs (Figure 6b,c). In contrast, the average number of tSCs per NMJ was 2.03 ± 0.13 in DMH‐specific *Sirt1* knockdown mice, and many NMJs had only one to two tSCs per NMJ, reflecting a decrease in the distribution of tSC numbers per NMJ. Nerve terminal abnormalities were increased (9.4 ± 3.1% with partially and fully denervated NMJs, *p* <* *.05, Figures 5g–h and 6a) similar to age‐related NMJ innervation anomalies. DMH‐specific *Sirt1* knockdown, however, did not induce postsynaptic AChR fragmentation (Figures 5 and 6a), in contrast to age‐related AChR fragmentation. In summary, DMH‐specific *Sirt1* knockdown resulted in more abnormalities of NMJ components. While some of the tSC morphologic changes differed from those observed in aged mice, the increased frequency of NMJ abnormalities is similar to the aged NMJ.

### 2.5 Muscle force did not correlate with age or Sirt1 expression

Tetanic muscle force assessments are summarized for all genotypes in Table 2. Tetanic muscle force was assessed at 3, 14, 17, and 25 months of age in the EDL muscles of WT mice. While there was a significant decrease in force between 3 and 14 months of age (27.53 N/cm^2^ vs. 17.42 N/cm^2^, *p *< .05), muscle force measurements did not remain low for 17‐ and 25‐month‐old mice (25.34 and 21.18 N/cm^2^, respectively; *p *> .05). No differences in muscle force were noted between BRASTO mice and their age‐matched controls at 16 months of age. In the EDL muscles of DMH‐specific *Sirt1* knockdown mice and their age‐matched controls, tetanic muscle force varied widely among animals, ranging from 15.4 to 40.2 N/cm^2^ (average 26.57 ± 6.21 N/cm^2^) in DMH‐specific *Sirt1* knockdown mice compared to forces ranging from 17.3 to 32.9 N/cm^2^ (average 23.39 ± 6.04 N/cm^2^) in controls (*p *> .05).

**Table 2:**
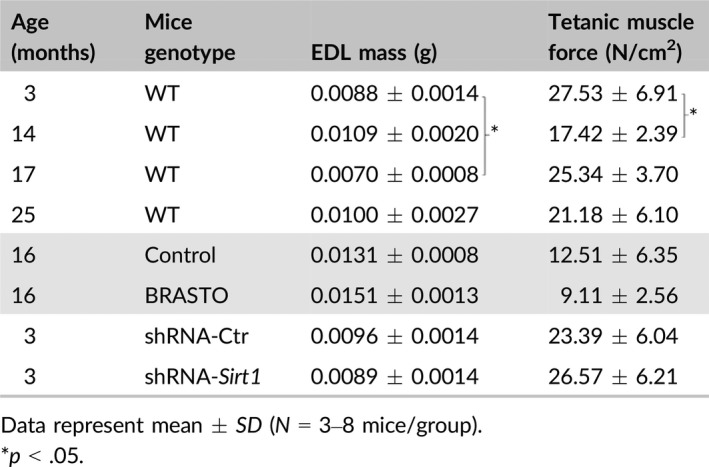
Tetanic muscle force measurements in EDL muscle

### 2.6 Sympathetic innervation is present at the NMJ and increased in BRASTO mice

**Figure 7:**
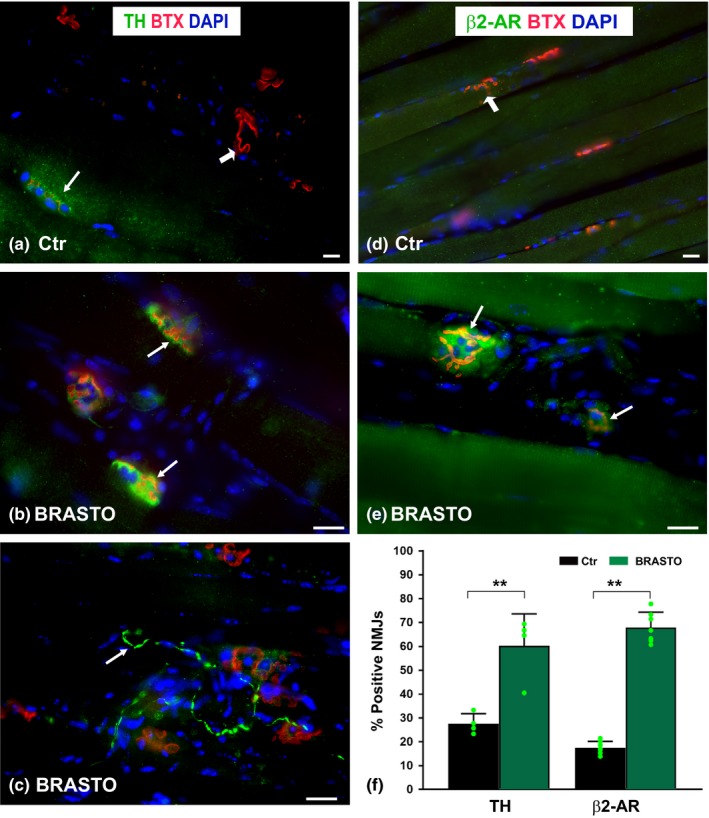
Aged (20‐month‐old) BRASTO mice with more youthful NMJs show increased sympathetic NMJ innervation (b, c, e, f) compared to NMJs in age‐matched Ctr (a, d, f) mice. Sternomastoid muscles are stained with antityrosine hydroxylase (TH, green, *arrows*, a–c) and anti‐β2‐adrenoreceptor (β2‐AR, *arrows*, d, e). Postsynaptic endplates (AChRs) and nuclei are labeled with BTX (red, *arrowheads*) and DAPI (blue), respectively. The percentages of TH‐ or β2‐AR‐positive NMJs are shown in panel (f) and are greater in BRASTO mice compared to controls. Scale bar = 20 μm. Data ± *SD*; each green dot represents average value calculated per animal; **p < .01

We demonstrated that BRASTO mice have a more youthful NMJ morphology, whereas DMH‐specific *Sirt1* knockdown mice display a more aged NMJ morphology. We hypothesized that the systemic effect of hypothalamic Sirt1 is mediated by the sympathetic nervous system. To assess the presence of sympathetic innervation near the NMJ, we stained SM muscles for the sympathetic markers tyrosine hydroxylase (TH), β2‐adrenoreceptor (β2‐AR), and neuropeptide Y (NPY) in aged (20‐month‐old) BRASTO mice and their age‐matched controls (Figure 7). All three markers demonstrated near colocalization with BTX staining in BRASTO mice, indicating the presence of sympathetic innervation at the NMJ. Minimal low‐intensity sympathetic staining was observed in aged control mice (Figure 7a,d). In contrast, more distinct sympathetic staining was noted at NMJs of BRASTO mice compared to control mice (Figure 7b,c,e). In addition, TH‐ (Figure 7c) and NPY‐ (data not shown) stained axons innervated, or were adjacent to, the NMJs (Figure 7c). Quantitative analyses showed more than double the percentage of TH‐positive and triple the percentage of β2‐AR‐positive NMJs in BRASTO mice compared to control mice (Figure 7f). A total of three β2‐AR antibodies were tested (two monoclonal and one polyclonal), and all showed similar results. This increased percentage of β2‐AR‐positive NMJs appears to be qualitatively consistent with higher mRNA expression levels of β2‐AR and higher cAMP levels in aged BRASTO skeletal muscle, compared to those in age‐matched control skeletal muscle (Satoh et al., 2013). NPY staining also showed similar colocalization to the NMJs (data not shown). Therefore, increased sympathetic NMJ innervation was observed with increased *Sirt1* function in the hypothalamus.

### 2.7 Sirt1 plays a protective role against age‐associated NMJ morphological abnormalities in the SM muscle

**Figure 8:**
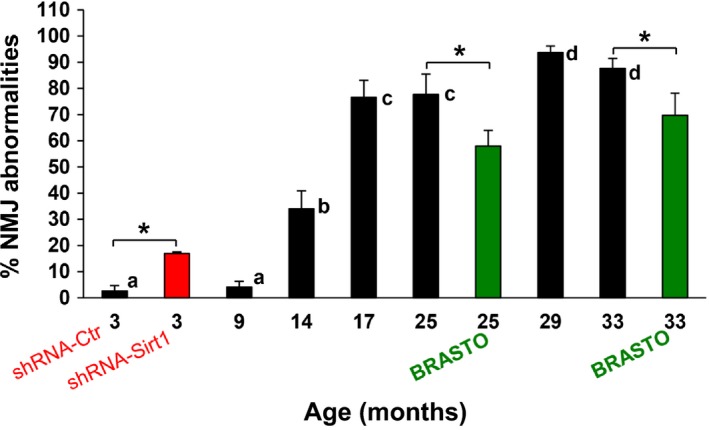
Sirt1 plays a protective role against age‐associated NMJ morphological abnormalities in the sternomastoid muscle. Percentages of NMJ abnormalities (≥1 in any of the 3 NMJ components) are shown for WT, BRASTO, and DMH‐specific *Sirt1* knockdown (shRNA‐*Sirt1*) mice at differing ages. Young adult mice (age 3 months) with DMH‐specific *Sirt1* knockdown (red bar) display a more aged NMJ morphology, with a significantly greater percentage of NMJ abnormalities than age‐matched controls (shRNA‐Ctr). BRASTO mice (ages 25 and 33 months, green bars) have a more youthful NMJ morphology, with significantly fewer NMJ abnormalities, than age‐matched controls. Data ± *SD*; *p < .05; different letter indicates significant difference (for 3‐, 25‐ and 33‐month‐old mice)

Lastly, we compared NMJ abnormalities in WT, BRASTO, and DMH‐specific *Sirt1* knockdown mice throughout all aged we examined (Figure 8). Clearly, central overexpression of *Sirt1* in aged BRASTO mice results in a more youthful (approximately 10 months younger) NMJ morphology, compared to age‐matched controls. Contrarily, *Sirt1* knockdown in the DMH of young mice results in a more aged (approximately 9–10 months older) NMJ morphology. Thus, these findings indicate that hypothalamic Sirt1 plays a critical role in protecting NMJ structures during the process of aging.

## 3 DISCUSSION

The aging process is associated with progressive loss of muscle mass, termed sarcopenia, which affects 13%–24% of humans under the age of 70 and 43%–60% of people over the age of 80 (Baumgartner et al., 1998). Neuromuscular dysfunction is a likely etiology of sarcopenia (Pannerec et al., 2016). Age‐associated changes occur not only in muscle and nerve, but also at the interface between the two, the NMJ. Age‐related morphologic NMJ changes have been previously described (Balice‐Gordon, 1997; Boaro et al., 1998; Chai, Vukovic, Dunlop, Grounds & Shavlakadze, 2011; Deschenes et al., 2010; Jang & Van Remmen, 2010; Kawabuchi et al., 2001; Luff, 1998; McMullen & Andrade, 2009; Smith & Chapman, 1987; Steinbach, 1981; Wernig & Herrera, 1986), and NMJ disruption has been identified as a driver of sarcopenia in a rodent model (Ibebunjo et al., 2013). The majority of those studies, however, described changes in nerve terminals or AChRs. In this study, we conducted comprehensive quantification of the age‐associated changes of all three of the main NMJ components: tSCs, nerve terminals, and motor endplates. In addition, we demonstrated that hypothalamic Sirt1 signaling through sympathetic innervation is critical to counteract age‐associated NMJ decline.

Given their roles in synaptic function and maintenance, tSC alterations could contribute to declines in muscle function with advancing age. With progressive age, we noted decreased tSC numbers at the WT NMJ, with abnormalities in tSC contour, staining, and cytoplasmic processes. Our tSC data corroborate with those of others (Boaro et al., 1998; Chai et al., 2011; Kawabuchi et al., 2001; Ludatscher, Silbermann, Gershon & Reznick, 1985). By 27 months of age, tSC degeneration was noted in 35% of NMJs in mouse gastrocnemius in contrast to the absence of tSC degenerative changes in 6‐month‐old mice (Ludatscher et al., 1985). Additionally, tSCs have been noted to be thin, disorganized, and to only partially cover the endplates in advanced aged mice (Chai et al., 2011; Kawabuchi et al., 2001), similar to the tSC changes we noted in the DMH‐specific *Sirt1* knockdown mice in this study. The functional sequelae of loss of tSC structure and number are unknown. Age‐related tSC loss, migration, and disorganization have been hypothesized to be related to myofiber denervation (Chai et al., 2011; Connor, Suzuki, Lee, Sewall & Heisey, 2002). Connor et al. (2002) noted extension of tSC cytoplasmic processes in 24‐month‐old rats in a manner similar to that seen after denervation (Connor et al., 2002), and Kawabuchi et al. (2001) noted similar findings in 24‐month‐old mice (Kawabuchi et al., 2001). Whether muscle denervation is a cause or effect of tSC changes, however, has not been conclusively demonstrated.

Nerve terminal morphology was also impacted by age in WT animals. Nerve terminals showed less branching with advancing age, and NMJ innervation declined with age. Similar morphological changes were noted in the DMH‐specific *Sirt1* knockdown mice. These findings suggest minimal motor nerve sprouting associated with remodeling, possibly resulting in permanent denervation for some muscle fibers. High numbers of denervated or partially innervated synapses with extremes of age have been previously described by others (Banker, Kelly & Robbins, 1983; Chai et al., 2011; Valdez, Tapia, Lichtman, Fox & Sanes, 2012).

Fragmentation of AChRs was noted in our study, and irregular and granular endplate fragmentation has been previously noted in aged mice (Balice‐Gordon, 1997; Chai et al., 2011; Pannerec et al., 2016; Valdez et al., 2012; Willadt, Nash & Slater, 2016). Pannerec et al. (2016) noted an increase in AChR fragmentation from 20% of NMJs affected in adult EDL muscle to 70% in sarcopenic muscle (Pannerec et al., 2016). Large amounts of motor endplate loss result in functional decline, but the functional impact of AChR fragmentation is less clear.

Alterations in nerve terminal and AChR morphology may be concerning for functional decline given their roles at the NMJ. The morphological changes noted in aged WT mice in our study, however, did not correlate with muscle force deficits. Similarly, we did not observe differences in muscle force between aged BRASTO and control mice and also between DMH‐specific *Sirt1* knockdown mice and controls. These findings may be the result of insufficient test sensitivity, insufficient time to assessment, or actual physiological compensation in the muscle. A compensatory mechanism fits with initial decline in muscle force that later improves to baseline levels. Similarly, others have reported no major age‐dependent physiological differences, despite age‐related morphological changes. The increased endplate fragmentation noted by Willadt et al. (2016) did not correlate with function; compound muscle action potential (CMAP) amplitude was stable with age (Willadt et al., 2016). Similarly, the absence of nerve terminals in 40% of NMJs in 34‐month‐old mice did not result in major physiological changes compared to young (8‐ to 12‐month‐old) mice (Banker et al., 1983). Functional stability might result from compensatory mechanisms, such as motor unit enlargement or increased synaptic vesicle release (Robbins, 1992), or from incomplete NMJ affliction with age‐related changes, as greater than 80% denervation is required to impart a functional deficit in muscle force (Gordon, Yang, Ayer, Stein & Tyreman, 1993). The aging synapse, therefore, is highly compensated rather than in a progressive state of deterioration from youth.

Importantly, we demonstrated that changes in central *Sirt1* expression counteract age‐associated NMJ decline in skeletal muscle. Brain‐specific overexpression of *Sirt1* results in a more youthful NMJ phenotype compared to age‐matched controls with respect to all three of the main NMJ components: tSCs, nerve terminals, and AChRs. Conversely, DMH‐specific knockdown of *Sirt1* resulted in more aged tSC and NMJ morphologies (Figure 8). Of the NMJ components, tSCs showed the biggest effect from *Sirt1* knockdown within the DMH, suggesting that tSCs may be early harbingers of systemic or environmental change. This hypothesis requires further investigation. Interestingly, overexpression of *Sirt1* in skeletal muscle does not delay metabolic age‐related changes (White et al., 2013). Together, these results implicate central *Sirt1* expression within the hypothalamus, rather than local or peripheral *Sirt1* expression, as a modifier of tSC and NMJ morphology.

We hypothesized that the sympathetic nervous system mediates the effects of central *Sirt1* expression on NMJ morphology. Impaired sympathetic neural function is associated with several age‐related systemic changes (Santulli & Iaccarino, 2013), and systemic physiological improvements seen in BRASTO mice result from sympathetic stimulation (Satoh et al., 2013). In addition, the sympathetic nervous system is present within skeletal muscle of young adult mice (Khan et al., 2016; Rudolf et al., 2013) and may modify muscle contraction, perfusion, and metabolism (Rudolf et al., 2013). Sympathetic innervation, as indicated by TH staining, was expressed in 96% of NMJs in the mouse EDL and 91% of those in the soleus (Khan et al., 2016). This same study found that chemical sympathectomy resulted in decreased NMJ size, complexity, and electrophysiologic values that were rescued by sympathicomimetic agents. Another sympathetic receptor that mediates responses to catecholamines, the β2‐AR, is found extensively throughout the body. While all three β‐adrenergic receptor types are present in skeletal muscle, there is a 10‐fold increased proportion of the β2 isoform in skeletal muscle (Santulli & Iaccarino, 2013). An age‐related decline in responsiveness to β2‐ARs has been proposed (Ford, Dachman, Blaschke & Hoffman, 1995), and β2 receptor agonists have reversed age‐related muscle weakness and wasting in rats (Ryall, Plant, Gregorevic, Sillence & Lynch, 2004). Because of the evidence supporting sympathetic innervation contributing to more youthful systemic physiology and NMJ morphology and function, we evaluated the presence of sympathetic innervation in our models via TH, β2‐AR, and NPY staining. We showed colocalization of all of these markers with AChRs, confirming sympathetic innervation at the NMJ. Interestingly, quantification of sympathetic NMJ innervation showed a twofold increase in the NMJs (Rudolf et al., 2013) staining for TH and a threefold increase in NMJs staining for β2‐AR in BRASTO mice compared to age‐matched controls. These data suggest that the mechanism linking the more youthful NMJ morphology associated with central *Sirt1* overexpression is sympathetically mediated. Manipulation of sympathetic innervation and activity is required in future studies to elucidate this mechanism.

Our study demonstrates degenerative changes in the three main NMJ components: tSCs, nerve terminals, and AChRs with advancing age. We further illustrate brain‐specific *Sirt1* overexpression resulting in more youthful morphologies of all NMJ components, as well as more aged NMJ morphologies with DMH‐specific *Sirt1* knockdown. The NMJ effects seen with manipulation of hypothalamic *Sirt1* expression confirm the importance of Sirt1 in delaying age‐related changes in skeletal muscle. The mechanisms linking central *Sirt1* expression with muscle structure remain to be elucidated, but the data from this study suggest an association with sympathetic innervation.

## 4 CONCLUSIONS

Age‐associated morphologic changes occur in all NMJ components, including tSCs, nerve terminals, and AChRs, with ~80% of NMJs exhibiting synaptic abnormalities by 17 months of age. Brain‐specific *Sirt1* overexpression results in more youthful‐appearing NMJs, and DMH‐specific *Sirt1* knockdown effects a more aged NMJ morphology. Differences in NMJ morphology due to *Sirt1* central expression are associated with changes in sympathetic innervation at the NMJ. We conclude that central Sirt1 expression protects against age‐related decline in skeletal muscle, mediated via the NMJ and sympathetic nervous system.

## 5 EXPERIMENTAL PROCEDURES

### 5.1 Mice

The analyses were performed in wild‐type (WT, C57BL/6J) and *S100‐GFP* transgenic mice at 3, 9, 14, 17, 20, 25, 29, and 33 months of age. *S100‐GFP* mice (generous gift from Dr. Susan Mackinnon, Washington University School of Medicine, St. Louis, MO) utilize the S100B promoter to drive GFP expression in all glial cells (Feng et al., 2000). We also used two types of Sirt1 mice: transgenic mice (7‐, 16‐, 20‐, 25‐ and 33‐month‐old) where brain‐specific *Sirt1* was overexpressed (BRASTO mice (Satoh et al., 2010, 2013) and shRNA‐*Sirt1*‐injected mice (3‐month‐old) where *Sirt1* was knocked down in the DMH using lentivirus and the stereotactic injection procedure described previously (Satoh et al., 2013)). The BRASTO mice with an HA‐tagged *Sirt1* transgene driven by the mouse prion promoter were backcrossed to C57BL/6J for 8 to 10 generations (Satoh et al., 2010). Mice in the aging cohorts were carefully inspected every day for all experiments, and both sexes were used for experiments. All aged animals studied had a normal appearance, with no signs of motor paralysis, muscle weakness, or any other type of visible disorder.

All experimental animals were housed in a central animal facility and were maintained pre‐ and postoperatively in strict accordance with the National Institutes of Health guidelines and according to protocols approved by the Division of Comparative Medicine at Washington University School of Medicine.

### 5.2 Muscle immunofluorescence staining and NMJ imaging

After anesthesia, animals were transcardially perfused with 0.1 m phosphate‐buffered saline (PBS, pH = 7.4) with 4% paraformaldehyde solution (Electron Microscopy Science, Hatfield, PA, USA) in PBS. Mouse SM muscle, which contains a relatively greater number of NMJs compared to other muscles, thereby providing an excellent model for NMJ evaluation, was selected for our studies. SM muscles from both sides of the neck were dissected and postfixed overnight at 4°C. For immunofluorescence labeling of NMJ components, muscles were processed as whole mounts (Love & Thompson, 1998) or as ~30‐μm‐thick longitudinal frozen sections, prepared after cryoprotection in 30% sucrose overnight at 4°C and embedding in OCT (Tissue‐Tek, Miles, Elkhart, IN, USA). All OCT specimens were stored at −80°C until use. Whole muscles or frozen sections were blocked and permeabilized first in the blocking buffer (2% normal goat serum, 2% Triton X‐100, 5 % bovine serum albumin in PBS) for 1 hr at room temperature followed by staining with the primary antibody overnight at 4°C. The following primary antibodies (Abs) were used: rabbit anti‐S100 (1:1,000; Dako North America, Via Real, Carpinteria, CA, USA) to visualize all Schwann cells (SCs), mouse antisynaptic vesicle protein 2 (SV2; 1:20; Developmental Studies Hybridoma Bank, Iowa City, IA, USA), rabbit antineurofilament 200 (NF200, 1:500; Millipore Sigma, St. Louis, MO, USA), mouse and rabbit anti‐β2‐adrenergic receptors (β2‐AR; 1:200; Santa Cruz Biotechnology, Inc., Dallas, TX, USA), rabbit monoclonal antineuropeptide Y (NPY; 1:50; Cell Signaling Technology, Inc., Boston, MA, USA), and rabbit antityrosine hydroxylase (TH; 1:50; EMD Millipore Corporation, Temecula, CA, USA). After rinsing in washing buffer (PBS with 2% Triton X‐100), sections (or muscles) were incubated with goat anti‐rabbit IgG‐Alexa Fluor 488 or goat anti‐rabbit IgG‐Alexa Fluor 594, and goat anti‐mouse IgG‐Alexa Fluor 488 (1:1,000; Invitrogen‐Molecular Probes, Carlsbad, CA, USA). After further rinsing in washing buffer, tissues were incubated with Alexa Fluor 555‐α‐BTX (1:1,000; Molecular Probes, Eugene, OR, USA), which binds specifically to AChRs in the postsynaptic membrane, thereby marking synaptic sites. Control sections (either no primary or no secondary antibody) were included in every type of staining to test for nonspecific staining and autofluorescence. Slides were prepared using Vectashield mounting medium with DAPI (Vector Laboratories, Inc. Burlingame, CA, USA) to label nuclei.

### 5.3 NMJ analysis and quantification

NMJ analyses were performed on whole mounts and frozen sections of the SM muscles (Love & Thompson, 1998; Valdez et al., 2010). To facilitate whole mount imaging, the entire SM muscle was flat mounted in Vectashield mounting medium with a coverslip. Morphologic changes between young adult and aging mice were analyzed in three major NMJ components: tSCs (S100 Ab labeling), the presynaptic nerve terminals (NF200/SV2 Abs labeling), and postsynaptic AChRs on the motor endplate (α‐BTX labeling). The whole mount muscle and muscle sections were imaged using an Axio Imager M2 fluorescent microscope (Zeiss, Thornwood, NY, USA). High‐resolution confocal images were obtained using a FluoView FV1000 confocal microscope (Olympus, Center Valley, PA, USA). Sequential capture was used to separate the green and red channels in order to prevent crosstalk between dyes, and Z‐serial images were collected with a 20×, 40×, and 63× objectives. Maximum intensity projections were obtained by epifluorescence scope or Olympus FluoView software and saved as TIFF and oib files. Whenever possible, image acquisition parameters remained constant. Images were viewed and analyzed using NIH ImageJ (http://rsb.info.nih.gov/iJ/), and figures were prepared using Excel, Adobe Photoshop CC 2015, and Adobe Illustrator CC 2015 system (Adobe Systems, San Jose, CA, USA).

For quantitative analysis, muscle sections from three to nine animals from each age‐group were processed. At least 15 sections and at least six or more macroscopic fields of view/section were analyzed per muscle totaling >100 NMJs per muscle. The sections selected for staining were from different parts of the muscle, but more sections were analyzed from middle portion of the muscle where the NMJ distributions were nearly identical at any sectioning level. All quantitative analyses were confirmed via blinded evaluators.

The three cellular components lying in close apposition at the NMJs—tSCs with their processes, motor nerve terminals, and AChRs—were evaluated. Only NMJs with motor endplates lying *en face* in the major plane of section were selected for quantification. Muscles where antibody staining was too faint to quantify due to poor antibody penetration were excluded from further analysis.

The percent of NMJs with tSCs (cell bodies) and the number of tSCs present per NMJ were determined in images labeled with S100 Ab, α‐BTX, and DAPI (Love & Thompson, 1998). Because multiple DAPI‐labeled nuclei are located at the NMJ, tSC identification was performed with care. The confocal z‐stacks were manually scanned above and below the endplate to identify the number of DAPI‐positive nuclei corresponding to S100 staining.

NMJ innervation (mostly or completely apposed endplates, stained with NF200/SV2 Abs, α‐BTX, and DAPI) was determined by classifying each NMJ in a given field of view as fully innervated (nerve terminal completely overlies α‐BTX), partially innervated (nerve terminal only partially covers α‐BTX), or vacant (no nerve terminal overlying the endplate) (Valdez et al., 2012). To ensure that lack of nerve terminal staining is not simply due to poor antibody penetration, surrounding NMJs and endplates were carefully observed. Diffused and/or reduced intensity of endplate staining, caused by the lack of neuronal signal and subsequent AChR dispersal, is an indicator of denervation.

The morphological changes in AChRs stained with α‐BTX and DAPI were determined by classifying endplates as normal (AChRs form a continuous structure also described as pretzel‐like, showing five or fewer fragments), partially fragmented (AChRs with more than five fragments or one irregular structure), or fully fragmented (AChRs with patchy or granular distribution). Most NMJs in young adult mice contain fewer than five AChR islands (Li et al., 2011).

The presence of sympathetic innervation at the NMJs was analyzed in the SM muscle sections from 20‐month‐old BRASTO (*n* = 4) and control (*n* = 3) mice stained with antibodies against β2‐AR, NPY, and TH in combination with α‐BTX and DAPI.

### 5.4 Preparation of lentiviruses expressing shRNA‐Sirt1 and shRNA‐Ctr

For a loss‐of‐function approach, we used short hairpin RNA (shRNA) to knock down the endogenous expression of *Sirt1* from the DMH. shRNA‐*Sirt1* and control (shRNA‐Ctr) lentivirus were prepared in the Hope Center Viral Vectors Core at Washington University School of Medicine as described previously (Satoh et al., 2013). Lentivirus was produced by cotransfecting HEK293T cells with vectors containing the *firefly luciferase* or *Sirt1* shRNA via the calcium phosphate precipitation procedure (Li et al., 2010). Virus with titer of 3.6 to 4.3 × 10^8^ TU/ml was used for stereotactic injection (see below). Knockdown efficiency was tested using primary hypothalamic neuronal cultures. Total RNA was extracted using the Pure Link RNA Mini kit (Ambion by Life Technologies, Carlsbad, CA, USA). Quantitative real‐time RT–PCR was conducted using primer–probe combination obtained from Applied Biosystems TaqMan Gene Expression Assays (Life Technologies), and relative expression levels were calculated for *Sirt1* by normalizing to *Gapdh* levels and then to one of the WT control individuals as previously described (Satoh et al., 2013). Transcript level fold change was determined by the delta CT method. All measurements were made in triplicate. Student's t test was performed to compare fold change. Two separate lentivirus samples were made for two different injection experiments.

### 5.5 Stereotactic injection of lentivirus expressing Sirt1 or firefly luciferase shRNA into the DMH

WT mice (3‐month‐old; *n* = 4 mice/group/experiment; *n* = 2 experiments) were used for stereotactic injection of experimental (shRNA‐*Sirt1*) or control (shRNA‐Ctr) lentivirus. The injection was performed under aseptic technique at the Animal Surgery Core at Washington University School of Medicine according to the method previously described in detail (Satoh et al., 2013). Immediately after the injection, animals were allowed to recover in a temperature regulated incubator (32°C) until fully awake and then were transferred to an isolated animal room for 72 hr and maintained without disturbance. All injected mice had 3 weeks to fully recover before being used for tissue collection. All procedures were in compliance with NIH Guidelines for Research Involving Recombinant DNA Molecules, and all animal usage methods were approved by the Division of Comparative Medicine and Institutional Animal Committee at Washington University.

### 5.6 Tetanic muscle force testing

Young adult WT, aged WT, DMH‐specific *Sirt1* knockdown mice, and BRASTO with their control mice were used for measuring the evoked compound action potential (cMAP) in the extensor digitorum longus (EDL) muscle upon electrical stimulation of the sciatic nerve as described previously in detail (Santosa et al., 2013). Animals were anesthetized and immobilized in an automated functional station (FASt System, Red Rock laboratories, St. Louis, MO, USA). Twitch contractions were used to estimate the optimal stimulus amplitude and optimal muscle length for isometric force production in the EDL muscle, with the distal muscle tendon fixed to a 5 Newton (N) load cell. The EDL, instead of SM, muscle was utilized as it has a long tendon, which is required to facilitate the fixation to the load cell. Tetanic contractions were recorded at increasing frequencies of stimulation (5–200 Hz), allowing 2‐min intervals between stimuli to prevent muscle fatigue. Maximum isometric tetanic force was automatically calculated from the resulting sets of recorded force traces. Following assessment, animals were euthanized, the dissected EDL muscle was weighed, and the tetanic specific muscle force (N/cm^2^) was calculated by dividing the absolute muscle force by the physiological muscle cross‐sectional area. A total of 3–8 animals per group were assessed.

### 5.7 Statistical analyses

All data are presented as the mean ± *SD*. Significance is shown as *p < .05. Quantifications were performed from at least three experimental groups in a blinded fashion unless otherwise noted. Statistical analyses were performed by two‐tailed, unpaired Student's *t* tests, or one‐way ANOVA followed by a Bonferroni post hoc test. All analyses were performed using Microsoft Excel, graphpad prism 6.00 (San Diego, CA, USA; http://www.graphpad.com), and spss (SPSS Inc., Chicago, IL, USA).

## CONFLICT OF INTEREST

The authors have no competing financial interests.

## AUTHOR CONTRIBUTIONS

ASW designed and supervised the study, performed data analysis, and wrote the manuscript. AS assisted with experiments and data analysis. KBS performed muscle force testing and assisted with manuscript preparation. SI assisted with study design and supervision. AJS performed experiments and data analysis, assisted with experimental design and supervision, and wrote the manuscript. All authors discussed data and provided critical revisions of the manuscript.
