# Supplementary figures and images for: Hypothalamic Sirt1 protects terminal Schwann cells and neuromuscular junctions from age‐related morphological changes
Source: Aging Cell. 2018 May 30;17(4):e12776. doi: 10.1111/acel.12776 (PMC6052483; doi:10.1111/acel.12776)

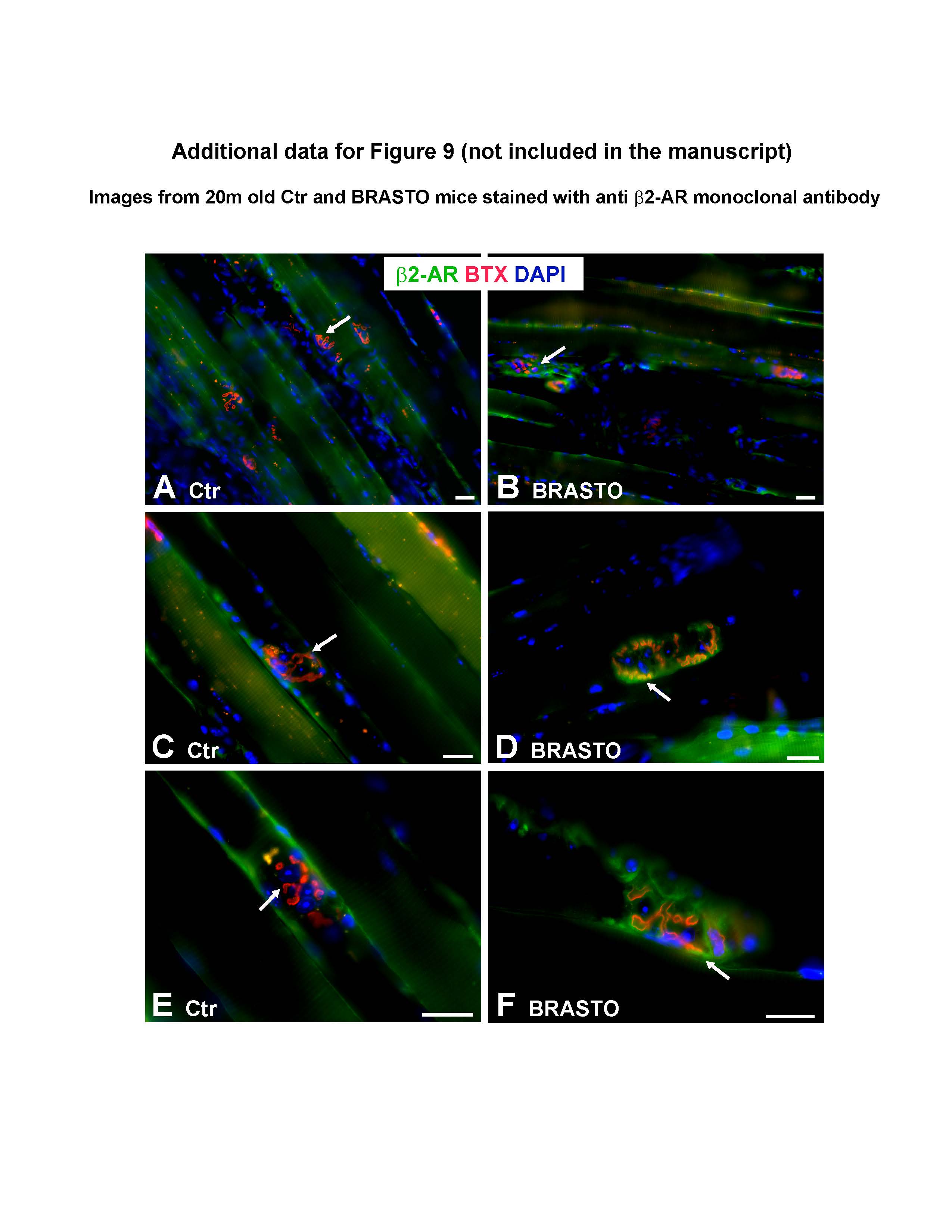

Supplement: Supplementary file 1 [file ACEL-17-na-s001.jpg]
